# Leakage correction improves prognosis prediction of dynamic susceptibility contrast perfusion MRI in primary central nervous system lymphoma

**DOI:** 10.1038/s41598-017-18901-x

**Published:** 2018-01-11

**Authors:** Yeon Soo Kim, Seung Hong Choi, Roh-Eul Yoo, Koung Mi Kang, Tae Jin Yun, Ji-hoon Kim, Chul-Ho Sohn, Sung-Hye Park, Jae-Kyung Won, Tae Min Kim, Chul-Kee Park, Il Han Kim

**Affiliations:** 1 Department of Radiology, Seoul National University Hospital, 101 Daehak-ro, Jongno-gu, Seoul, 03080 Korea; 2 Department of Radiology, Seoul National University College of Medicine, 103 Daehak-ro, Jongno-gu, Seoul, 110-799 Republic of Korea; 3 Department of Pathology, Seoul National University College of Medicine, Seoul, Korea; 4 Department of Internal Medicine, Cancer Research Institute, Seoul National University College of Medicine, Seoul, Korea; 5 Department of Neurosurgery, Biomedical Research Institute, Seoul National University College of Medicine, Seoul, Korea; 6 Department of Radiation Oncology, Cancer Research Institute, Seoul National University College of Medicine, Seoul, Korea

## Abstract

To evaluate whether the cerebral blood volume (CBV) measurement with leakage correction from dynamic susceptibility contrast perfusion weighted imaging can be useful in predicting prognosis for primary central nervous system lymphoma (PCNSL). 46 PCNSL patients were included and classified by radiation therapy (RT) stratification into RT (n = 30) and non-RT (n = 16) groups. The corresponding histogram parameters of normalized CBV (nCBV) maps with or without leakage correction were calculated on contrast-enhanced T1 weighted image (CE T1WI) or on fluid attenuated inversion recovery image. The 75^th^ percentile nCBV with leakage correction based on CE T1WI (T1 nCBVL_75%_) had a significant difference between the short and long progression free survival (PFS) subgroups of the RT group and the non-RT group, respectively. Based on the survival analysis, patients in the RT group with high T1 nCBVL_75%_ had earlier progression than the others with a low T1 nCBVL_75%_. However, patients in the non-RT group with a high T1 nCBVL_75%_ had slower progression than the others with a low T1 nCBVL_75%_. Based on RT stratification, the CBV with leakage correction has potential as a noninvasive biomarker for the prognosis prediction of PCNSL to identify high risk patients and it has a different correlation with the PFS based on the presence of combined RT.

## Introduction

Primary central nervous system lymphoma (PCNSL) is a rare primary brain cancer as an extra-nodal variant of non-Hodgkin lymphoma confined to the central nervous system with variable response to treatment and clinical outcomes^1–3^. With respect to the clinical outcome, immune deficiency is the only established risk factor for developing PCNSL^1,3^. In immunocompetent patients, the prognosis of PCNSL is highly variable^4^. Several reported clinical markers were associated with the prognosis such as age, Eastern Cooperative Oncology Group (ECOG) performance status, serum level of lactate dehydrogenase, cerebrospinal fluid protein concentration, and involvement of deep brain regions^5^. However, noninvasive biomarkers of the prognosis have continued to identify high risk groups at initial diagnosis to determine a personalized therapeutic strategy^6^. For immunocompetent patients with PCNSL, the treatment options include chemotherapy, radiation therapy (RT), and the combination of both modalities^1^. Although the cornerstone of therapy is a systemic treatment with intravenous high-dose methotrexate, there remains controversy about the role of RT^3^. For this reason, combined RT is not strongly recommended, but it is considerable whether there is its weighted benefit to be considered against the increased neurotoxicity risk^7,8^.

To stratify a personalized therapy and assess the response, noninvasive biomarkers of the prognosis should be quantitatively and serially measured. There have been several published reports suggestive of a significant correlation between nonconventional physiology-based magnetic resonance (MR) imaging modalities such as diffusion weighted images derived from apparent diffusion coefficient values and clinical outcomes of PCNSL treatment^9^. There have been relatively few studies of the effectiveness of dynamic susceptibility contrast perfusion weighted imaging (DSC PWI) derived normalized cerebral blood volume (nCBV) values in PCNSL that can assess the degree of tumor angiogenesis, and capillary permeability to assess the response to therapy compared to gliomas^10,11^.

In DSC PWI, the signal intensity time curve is described with the percentage of signal intensity recovery at the end of the first pass^12,13^. In the presence of contrast agent extravasation because of severely compromised blood brain barrier (BBB), the underlying kinetic model used in the perfusion weighted image that the contrast agent is contained in the intravascular space during the dynamic acquisition may not be valid^14^. Contrast agent leakage from the intravascular compartment to extravascular-extravascular space has to be corrected for accurate measurement of the cerebral blood volume (CBV). With BBB disruption, high permeability with contrast extravasation into the extravascular-extracellular space induces a T2* weighted signal intensity drop which causes CBV underestimation^15^.

Because there was a high degree of BBB disruption and thus a high vascular permeability in PCNSL, leakage correction may imply greater accuracy in the CBV measurement^16^. Therefore, the purpose of this study is to evaluate whether the CBV measurement with leakage correction from DSC PWI can be useful for the prognosis prediction of PCNSL.

## Results

### Patient population

Clinical parameters, including the age, sex, ECOG score, and interval duration between pretreatment MR examination and the date of first medical treatment, were summarized and are shown in Table 1. Forty-six patients were classified into two groups according to treatment with RT; 30 patients with combined RT referred to as the RT group (65% [30/46]) and 16 patients without combined RT referred to as the non-RT group (35% [16/46]). The RT group consisted of 23 patients with a progression free survival (PFS) < 3 years designated short PFS subgroup, and 7 patients with a PFS ≥ 3 years as the long PFS subgroup. The non-RT group consisted of 7 patients with PFS < 1 year as the short PFS subgroup and 9 patients with a PFS ≥ 1 year as the long PFS subgroup (Table 1).

**Table 1:** Clinical characteristics in 46 patients with stratification of the RT group and PFS subgroup.

	Group and subgroup			
	RT		non-RT	
	Short PFS (<3 years)	Long PFS (≥3 years)	Short PFS (<1 year)	Long PFS (≥1 year)
Total patients (Male:Female)	23 (12:11)	7 (4:3)	7 (4:3)	9 (5:4)
*p*-value	0.8258		0.9535	
Median age (range)	54 (37–84)	50 (40–62)	66 (41–78)	63 (39–70)
*p*-value	0.1056		1.0000	
ECOG (grade)				
0–1:2–5 (number)	14:9	5:2	2:5	7:2
*p*-value	0.6298		0.1913	
Median interval duration (range)	17 (4–44)	11.5 (2–17)	13 (10–25)	13 (3–41)
*p*-value	0.0754		0.8735	

### Comparison analysis between the short PFS and long PFS subgroups in the RT group

#### Comparison of histogram parameters

**Figure 1:**
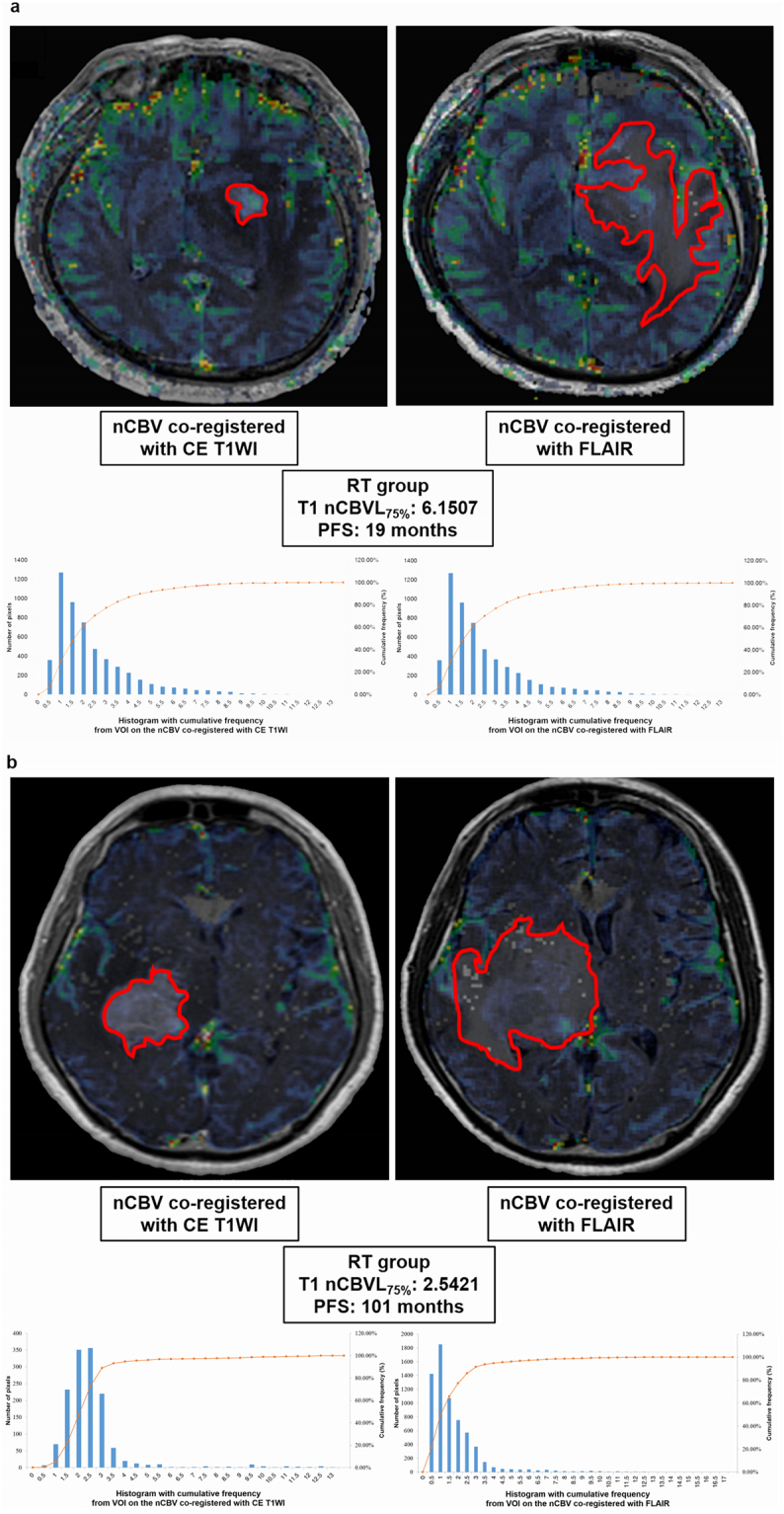
Representative cases of the short PFS and long PFS subgroups of the RT group. There were nCBV maps with leakage correction co-registered with CE T1WI (upper left) and nCBV map co-registered with FLAIR (upper right). Histogram and cumulative histogram of all pixel values of total VOIs, such as an enhancing lesion on CE-T1WI (lower left) and hyperintense lesion on FLAIR (lower right), were obtained from the aforementioned nCBV co-registration images. (**a**) nCBV maps with leakage correction and histograms in a 38-year-old male patient with a short PFS in the RT group. The T1 nCBVL_75%_ was 6.15 and PFS was 19 months. (**b**) nCBV maps with leakage correction and histograms in a 68-year-old male patient with a long PFS in the RT group. The T1 nCBVL_75%_ was 2.54 and PFS was 101 months.

Regarding the nCBV values with or without leakage correction, the result of the comparison analysis is shown on Supplementary Table 1. Among the analyzed values, the T1 normalized cerebral blood volume with leakage correction (nCBVL) _mean_, T1 nCBVL_75%_, T1 nCBVL_90%_, and T1 normalized cerebral blood volume without leakage correction (nCBVnL) _75%_ had significant differences between the short and long PFS subgroups (*P* < 0.05) (Supplementary Table 1). Among the aforementioned statistically significant variables, T1 nCBVL_75%_ had the highest value of the area under the receiver operating characteristic (ROC) curve (0.795, 95% confidence interval (CI), 0.609–0.920) (*P* < 0.05). The cutoff value of 5.3250 exhibited prediction rates of 65.22% (15/23) and 100% (7/7) for the short and long PFS subgroups, respectively. The multivariate logistic regression analysis showed that the T1 nCBVL_75%_ was the only independent variable for predicting the short and long PFS (*P* < 0.05). Then, 30 patients were stratified based on the value of T1 nCBVL_75%._ The classification cutoff value of T1 nCBVL_75%_ was 5.3250 and 15 patients had a T1 nCBVL_75%_ value ≤ 5.3250 and other 15 patients had a T1 nCBVL_75%_ value > 5.3250 (Fig. 1).

#### Comparison of total volume of interest (VOI)

The mean value of the total VOI based on the contrast-enhanced T1 weighted image (CE T1WI) and fluid attenuated inversion recovery (FLAIR) did not have significant difference between PFS subgroups in the RT group, respectively (Supplementary Table 1).

#### Comparison of RT modalities

With the respect to RT modalities, whole brain RT or involved field RT, which the patients in the RT group underwent, there was no significant correlation between the short PFS and long PFS subgroups (*P* > 0.05).

### Comparison analysis between the short PFS and long PFS subgroups in the non-RT group

#### Comparison of histogram parameters

**Figure 2:**
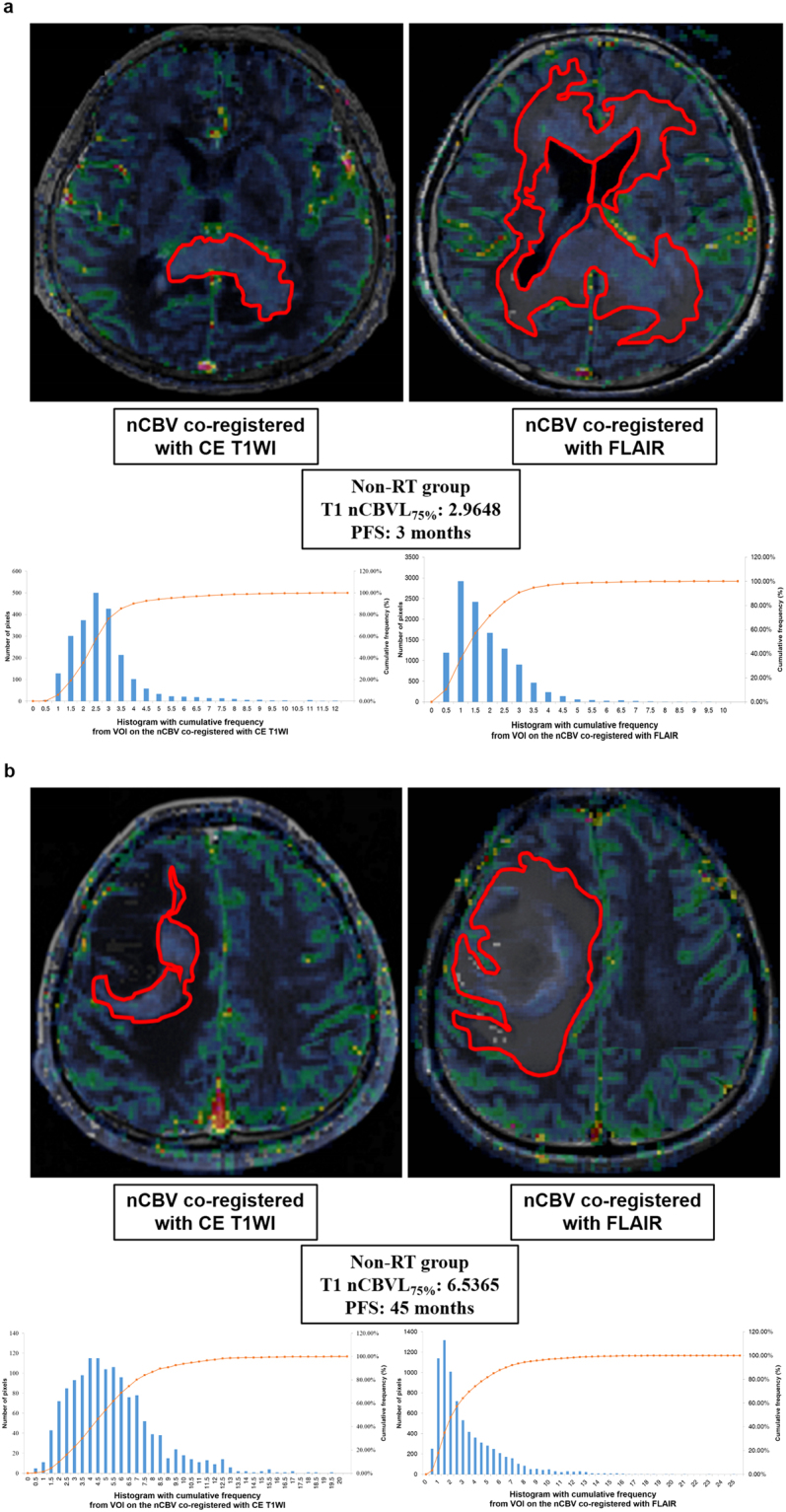
Representative cases of the short PFS and long PFS subgroups of the non-RT group. nCBV maps with leakage correction co-registered with CE T1WI (upper left) and FLAIR (upper right). Histogram and cumulative histogram of all pixel values of total VOIs, such as an enhancing lesion on CE T1WI (lower left) and hyperintense lesion on FLAIR (lower right), were obtained from the aforementioned nCBV co-registration images. (**a**) nCBV maps with leakage correction and histograms in an 81-year-old female patient with a short PFS of the non-RT group. The T1 nCBVL_75%_ was 2.96 and PFS was 3 months. (**b**) nCBV maps with leakage correction and histograms in a 44-year-old male patient with a long PFS of the non-RT group. The T1 nCBVL_75%_ was 6.53 and PFS was 45 months.

The result of the comparison analysis of the non-RT group is shown in Supplementary Table 2. The T1 nCBVL_mean_, T1 nCBVL_75%_, and T1 nCBVL_90%_ were significantly different between the short and long PFS subgroups (*P* < 0.05). Sixteen patients from the non-RT group were also stratified by T1 nCBVL_75%_ values with the highest value of the area under the ROC curve (0.841, 95% CI, 0.576–0.972), but the cutoff value of T1 nCBVL_75%_ for classification was 4.2243. The cutoff value of 4.2243 exhibited prediction rates of 100% (7/7) and 66.67% (6/9) for the long and short PFS subgroups, respectively. The multivariate logistic regression analysis showed that the T1 nCBVL_75%_ was the only independent variable for the prediction of short and long PFS (*P* < 0.05). There were 9 patients with a T1 nCBVL_75%_ value ≤ 4.2243 and another 7 patients with a T1 nCBVL_75%_ value > 4.2243 (Fig. 2).

#### Comparison of total VOI

Like in the RT group, the mean value of the total VOI based on the CE T1WI and FLAIR did not have significant difference between PFS subgroups in the non-RT group, respectively (Supplementary Table 2).

### Leave-one-out cross-validation (LOOCV) test in both RT and non-RT group

Results of LOOCV test are shown on Supplementary Table 3. In RT group, the average accuracy was 80.61% to predict prognosis based on the T1 nCBVL_75%_ cut-off value. The accuracy was 82.61% based on population data of RT group. In the same way, the average accuracy of LOOCV test was 83.33% in non-RT group which is the same value compared with that of population data.

### Survival analysis and Cox analysis in both RT and non-RT group

**Figure 3:**
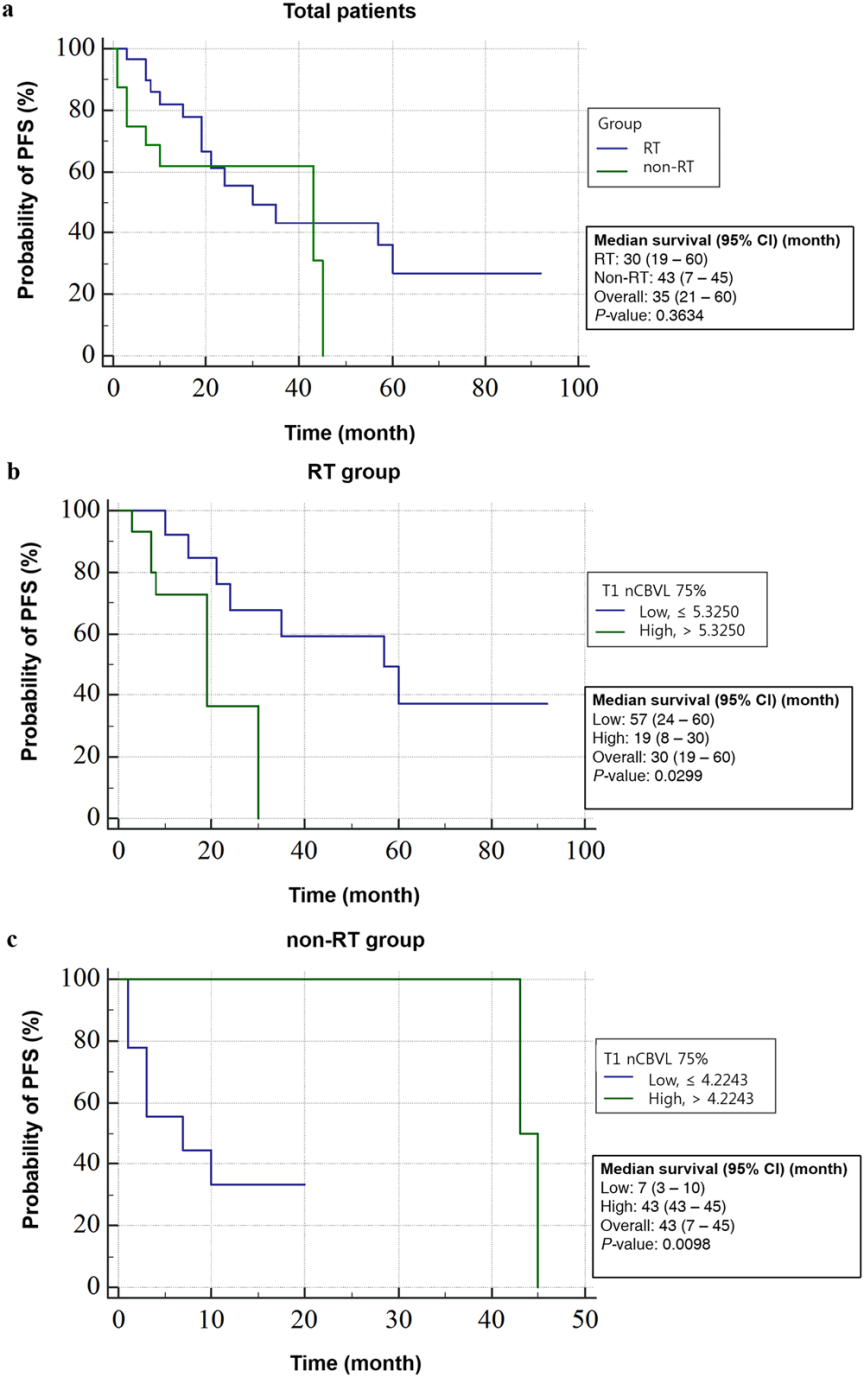
Kaplan-Meier survival graphs according to presence of RT or value of T1 nCBVL_75%_ obtained from pretreated DSC PWI. (**a**) Probability of the PFS based on the RT stratification is shown. The median PFS of the RT group patients (blue line) was 30 months ([95% CI, 19–60]) and that of the non-RT group patients (green line) was 43 months ([95% CI, 7–45]). This graph showed that there was no significant survival difference between the two groups (*p* –value = 0.3634, log-rank test). (**b**) In the RT group, the probability of PFS based on T1 nCBVL_75%_ with a cutoff value of 5.3250 is shown. Patients with a low T1 nCBVL_75%_ value ≤ 5.3250 (blue line) had a 57-month median PFS ([95% CI, 24–60]). Patients with a high T1 nCBVL_75%_ value > 5.3250 (green line) had a median PFS of 19 months ([95% CI, 8–30]) (*p* –value = 0.0299, log-rank test). (**c**) In the non-RT group, the probability of PFS based on the T1 nCBVL_75%_ with a cutoff value of 4.2243 is shown. Patients with a low T1 nCBVL_75%_ value ≤ 4.2243 (blue line) had a median PFS of 7 months ([95% CI, 3–10]) Patients with a high T1 nCBVL_75%_ value > 4.2243 (green line) had a median PFS of 43 months ([95% CI, 43–45]) (*p* –value = 0.0098, log-rank test).

Results of Kaplan-Meier survival analysis are shown on Fig. 3. There is no significant difference in the PFS between the RT and non-RT groups (median, 30 [95% CI, 19–60] and 43 [95% CI, 7–45] months, respectively) (*P* > 0.05, log-rank test) (Fig. 3a). In the RT group, patients with a high T1 nCBVL_75%_ value > 5.3250 had earlier progression than the others with a low T1 nCBVL_75%_ value ≤ 5.3250 (*P* < 0.05, log-rank test) (Fig. 3b). The median PFS of the high T1 nCBVL_75%_ subgroup was 19 months ([95% CI, 8–30]) and that of the low T1 nCBVL_75%_ subgroup was 57 months ([95% CI, 24–60]). On the other hand, in the non-RT group, patients with a low T1 nCBVL_75%_ value ≤ 4.2243 had an increased risk of early progression with median PFS of 7 months ([95% CI, 3–10]) compared to patients with a low T1 nCBVL_75%_ value > 4.2243 who had a longer median PFS of 43 months ([95% CI, 43–45]) (*P* < 0.05, log-rank test) (Fig. 3c).

Despite the similar high value of the T1 nCBVL_75%_, the patients in the RT group (Fig. 1a) had a relatively shorter PFS than the patients in the non-RT group (Fig. 2b). On the other hand, in the case of a similar low value of the T1 nCBVL_75%_, the patients in the non-RT group (Fig. 2a) had relatively shorter PFS than the patients in the RT group (Fig. 1b).

Based on Cox regression analysis, T1 nCBVL_75%_ value was the most (*P* < 0.05, [95% CI, 0.0024–0.9924], exp(B) = 0.0486) important factor incorporated into the multivariate model only in non-RT group. In RT group, there is no significant parameter that impact on recurrence.

### Heat map of quantitative values of DSC PWI in all patient with RT stratification

**Figure 4:**
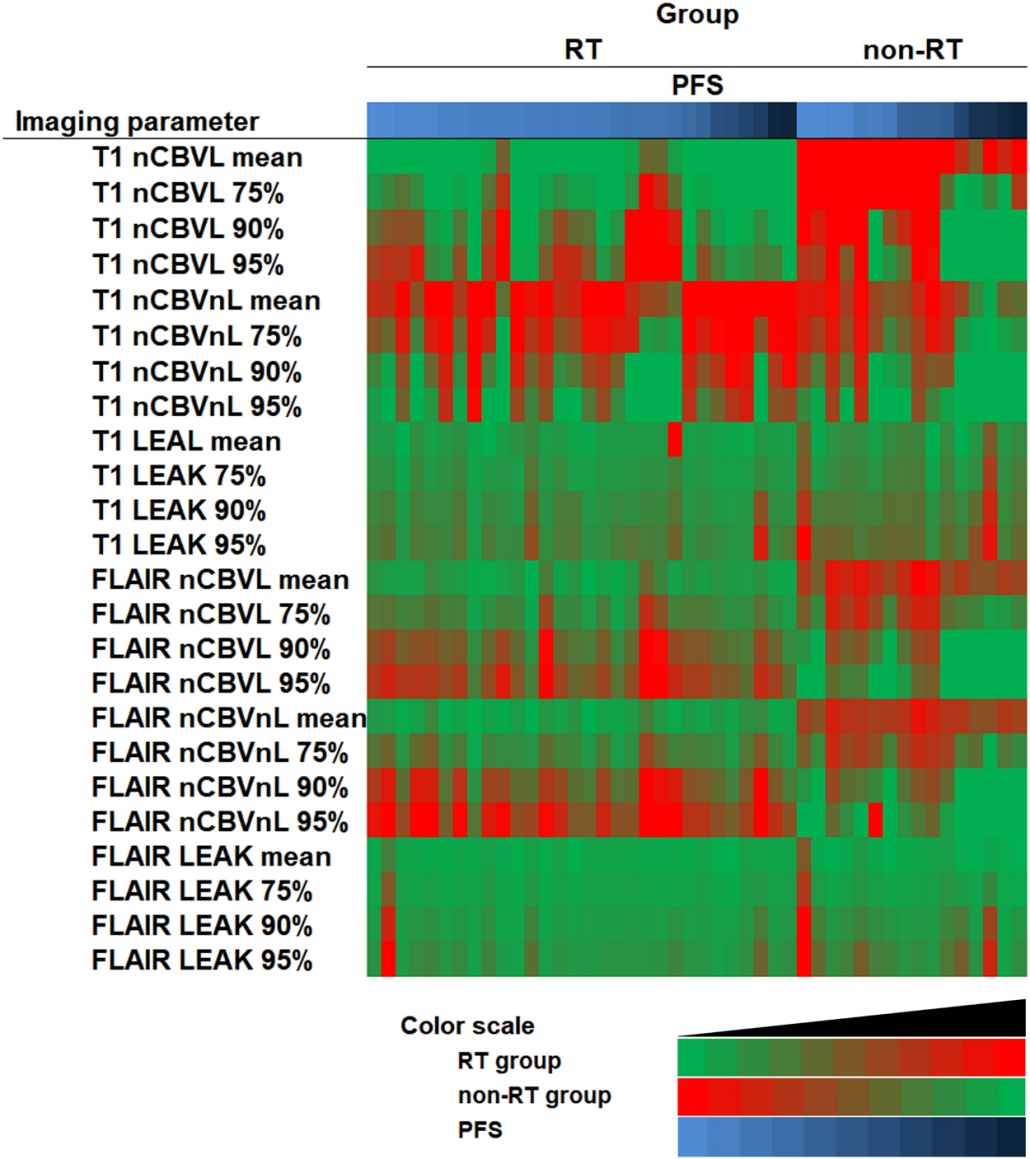
Heat map of quantitative values of DSC PWI in all patients with RT stratification. There are clusters of quantitative values of DSC PWI in all patients with RT and PFS stratification. In both groups, each patient was arranged in order of the PFS. Each value corresponding with each pixel was divided by the maximum value and its ratio ranged from 0 to 1. Each pixel color was determined by the aforementioned calculated ratio. Then, all variables are presented as representative colors based on calculated ratio and representative color scale. In the RT group, the mean and 75^th^, 90^th^, and 95^th^ percentiles of the nCBVL, nCBVnL, and LEAK derived from DSC PWI in conjunction with CE-T1WI and FLAIR are represented as red with relative high values and green with relative low values. On the other hand, in the non-RT group, the same statistics were represented as green with relative high values and red with relative low values. This heat map suggests that a high value of the T1 nCBVL represented as green had early progression with a shorter PFS in the RT group and low value of T1 nCBVL represented as green had early progression in the non-RT group. In conclusion, the relationship between T1 nCBVL and PFS differed in that there was negative correlation in the RT group and positive correlation in the non- RT group.

Data for individual values of the imaging parameters and the PFS according to the group were displayed as a graphical representation (Fig. 4).

## Discussion and Conclusions

In this study, we hypothesized that the nCBV values could have potential as noninvasive quantitative prognostic factors for the PFS and their relationship with the PFS could be different based on the treatment modalities. We found that the T1 nCBVL_75%_ values are the most predictive imaging biomarkers in immunocompetent patients with PCNSL. As expected, their relationship with the PFS was different according to the treatment modality of the patient. Specifically, in the RT group, patients with low T1 nCBVL_75%_ values have a significantly longer PFS than the others with high values. In contrast, in the non-RT group, patients with a high value of T1 nCBVL_75%_ value have a significantly longer PFS than the others with low values.

The DSC PWI estimates the tissue microvascular density by measuring the relative CBV values, which are a measurement of the microvascular blood volume in tumors, reflecting tumor angiogenesis and its energy metabolism^17^. Although there are relatively few studies, the value of relative CBV could be a non-invasive prognostic biomarker in PCNSL compared to glioma, and a low relative CBV is a novel risk factor for adverse prognosis in immunocompetent patients with PCNSL^6^. Our results are consistent with previous studies in some respects to the relationship between the nCBV value with leakage correction and PFS. The dynamic methods provide accurate measurement of the perfusion parameters only in the case of an intact BBB^18^. In consideration to the significant tendency for PCNSL to disrupt the BBB, leakage correction plays an important role to compensate for the tissue T1 and T2 variation because of the contrast leakage to the extravascular space^18^. In this study, the used dedicated image analysis software performed leakage correction based on both Weisskoff method applied for correcting the T1 effect of the contrast leakage^14,19^ and residue function based leakage correction method applied for both T1 and T2/T2* dominant leakage effects^19^. Consequently, the derived value of the nCBV with leakage correction might be accurate because both T1 and T2/T2* dominant leakage effects can be corrected.

Among various statistically significant variables obtained from histogram analysis, there were several variables to predict the prognosis in both groups (*P* < 0.05). This finding may suggest the relatively potential advantage of the cumulative histogram method analysis. Owing to its multifocal and infiltrative nature, it is difficult to select the representative lesion of PCNSL^20^. The use of the total voxel values of a tumor could identify the overall nature of the tumor and provide a better prognostic accuracy than a statistically comprehensive mean value^21,22^.

Based on the survival analysis in both groups, the relationship between the nCBV and PFS differed between the RT and non-RT groups. In the non-RT group, high nCBV values with leakage correction may estimate a longer PFS, which is probably because of their adequate patent vessel for delivery of chemotherapeutic agents^6^. As low nCBV values with leakage correction may suggest a relative lack of patent vessels delivering chemotherapeutic agent to the tumor bed, patients with a low nCBVL_75%_ value, suggestive of relative hypoxic environment, resisted treatment^6,23–25^. Consequently, low nCBVL_75%_ values correlated with a short PFS in the non-RT group.

On the other hand, in the RT group, we found that a low T1 nCBVL_75%_ value is related to a longer PFS. Considering the adverse relationship between the T1 nCBVL_75%_ and PFS in both the RT and non-RT groups, the major contributing factor may be suspected to be the radiation effect in the brain. Whole brain radiation therapy alone is insufficient for durable PCNSL control because of its limited efficacy as a single therapeutic modality because of its high risk of neurotoxicity, especially in elder patients^1^. If the PCNSL patient is tolerable for the chemotherapy, combined modality therapy or chemotherapy alone are favored rather than radiation therapy alone. Although a low nCBV value and leakage correction suggests hypoxia which plays a role in the resistance to chemotherapy and radiation in brain tumors^26^, the main therapeutic effect might be determined by the efficacy of the drug delivery in consideration to multi-agent chemotherapy as the current treatment of choice^1^. As chemotherapy is one of the major treatment modalities for PCNSL, drug delivery is the main challenge for achieving effective treatment^3^. There are few reports of the results of RT to induce BBB opening in patients with PCNSL. Although the BBB represents an insurmountable obstacle for many drugs^27^, there are a number of other barriers that inhibit systemically administered drug delivery to the tumor, such as the blood cerebrospinal fluid barrier, blood tumor barrier, and efflux mechanisms in drug transport^28^. The blood tumor barrier is one of the challenging obstacles. In addition, there are many physiological contributing factors that would induce the relatively poor delivery of drug to tumors, such as a heterogeneous blood supply, relatively long distances in the interstitium, cellular heterogeneities, and interstitial hypertension^29^. Drug delivery to tumor cells consists of a heterogeneous distribution of microvascular structure throughout the tumor interstitium^28^. As the intra-capillary distance increases for some causes, the vascular surface area decreases, reducing the trans-vascular exchange of blood-borne molecules^28^. As a representative cause of obstacles for drug delivery, the high interstitial tumor pressure and associated peri-tumoral edema increase the hydrostatic pressure in the adjacent normal brain parenchyma. As a result, the cerebral microvascular structure in perilesional area may be less permeable to drugs than the normal brain endothelium, leading to an exceptionally low extra-tumoral interstitial drug concentration^30^. Radiation induces ultrastructural changes of the blood capillaries of the brain and, consequently, accompanies capillary wall swelling, increases in the microcirculatory bed permeability, and perivascular edema with elevated interstitial pressure^31^. With a high nCBV value and leakage correction suggestive of increased tumor angiogenesis, there was an increased chance of interstitial hypertension induced by perivascular edema in the periphery and surrounding tissue for chemotherapeutic agents to overcome induction of difficult outward convection to diffuse into the tumor^28,29,32^. We postulate that the negative effect of increased perivascular pressure for drug delivery outweighs facilitated delivery by the RT-induced BBB disruption. Consequently, the efficient level of drug delivered to the tumor bed was decreased after RT because of the aforementioned reason. Many *in vivo* studies have documented that interstitial pressure is elevated in most solid tumors, and it presents a major obstacle to the transfer of drug from the blood into the tumor after RT^32,33^.

Based on results of Cox regression analysis, T1 nCBVL_75%_ was not predictable value only in RT group in spite of the aforementioned inverse relationship between its value and PFS. RT can induce irreversible changes such as fibrosis and consecutive obliteration of the small vessel because of the endothelial damage^34^. Those additional microenvironment change after RT would make it difficult to predict the prognosis based on the perfusion MR imaging. We believe that T1 nCBVL_75%_ is difficult to show statistical significance as a powerful predictor in the small number of patient group, because of the complex condition to be considered in radiated brain. Although the statistical methods cannot untangle a confounded condition in radiated brain, the overall aforementioned relationship between T1 nCBVL_75%_ and prognosis should be overlooked in RT group.

Our study has several limitations. First, limitations of this study include the low number of patients, the heterogeneity of the therapeutic protocols, and the absence of a replicative cohort. Second, the cutoff values used for PFS based subgroup classification are different between the RT and non-RT groups. As the number of the patients in the non-RT group was small in contrast to that in the RT group, we did not get the significant difference between the short and long PFS subgroups in the non-RT group with the same cutoff value, which was 3 years, in the RT group. To find the same cutoff value for the PFS subgroup classification between the RT and non-RT groups, studies with a large number of patients should be performed. Third, the retrospective study design required the use of heterogeneous treatment modalities that depend on each patient. As there were no definite established therapeutic guidelines, there were heterogeneous modalities, durations, and intervals of treatment in the enrolled patients. RT was not strongly recommended based on the medical evidence, but it was considered dependent on the performance status of each patient as well as the tumor response to chemotherapy, which was a relatively subjective finding based on the decision of the clinicians because there were no absolute objective criteria for the PCNSL treatment response. Fourth, we used imaging features from several kinds of MR scanners in our retrospective study, which might affect the present results. However, we used normalized quantitative values to minimize the potential difference due to MR scanner diversity.

In conclusion, the CBV with leakage correction as a predictable noninvasive biomarker for prognosis of PCNSL, has the potential to identify high-risk patients based on RT stratification and may be used to formulate a therapeutic strategy and estimate the response to therapy in PCNSL patients.

## Methods

This retrospective study was approved by our institutional review board, and informed consent was waived.

### Patient population

From January 2007 to April 2016, 130 patients with newly diagnosed PCNSL by surgical resection or biopsy, based on the World Health Organization criteria, were selected from the electronic medical records of our institution. The inclusion criteria were as follows: (a) histopathologically confirmed PCNSL; (b) immunocompetent state with negative immunodeficiency virus status; (c) absence of other lesions except the primary brain lesion based on other imaging modalities including computed tomography and fludeoxyglocuse - positron emission tomography scans; and (d) pretreated baseline conventional MR imaging with DSC PWI available for total volume analysis. Of 130 patients, 84 were excluded for the following reason: (a) inadequate pretreated MR imaging lack of DSC PWI appropriate for analysis (n = 63); (b) improper imaging data for analysis by imaging processing software (n = 3); (c) recurrent PCNSL (n = 6); and (d) other site involvement, except a primary brain lesion (n = 12).

**Figure 5:**
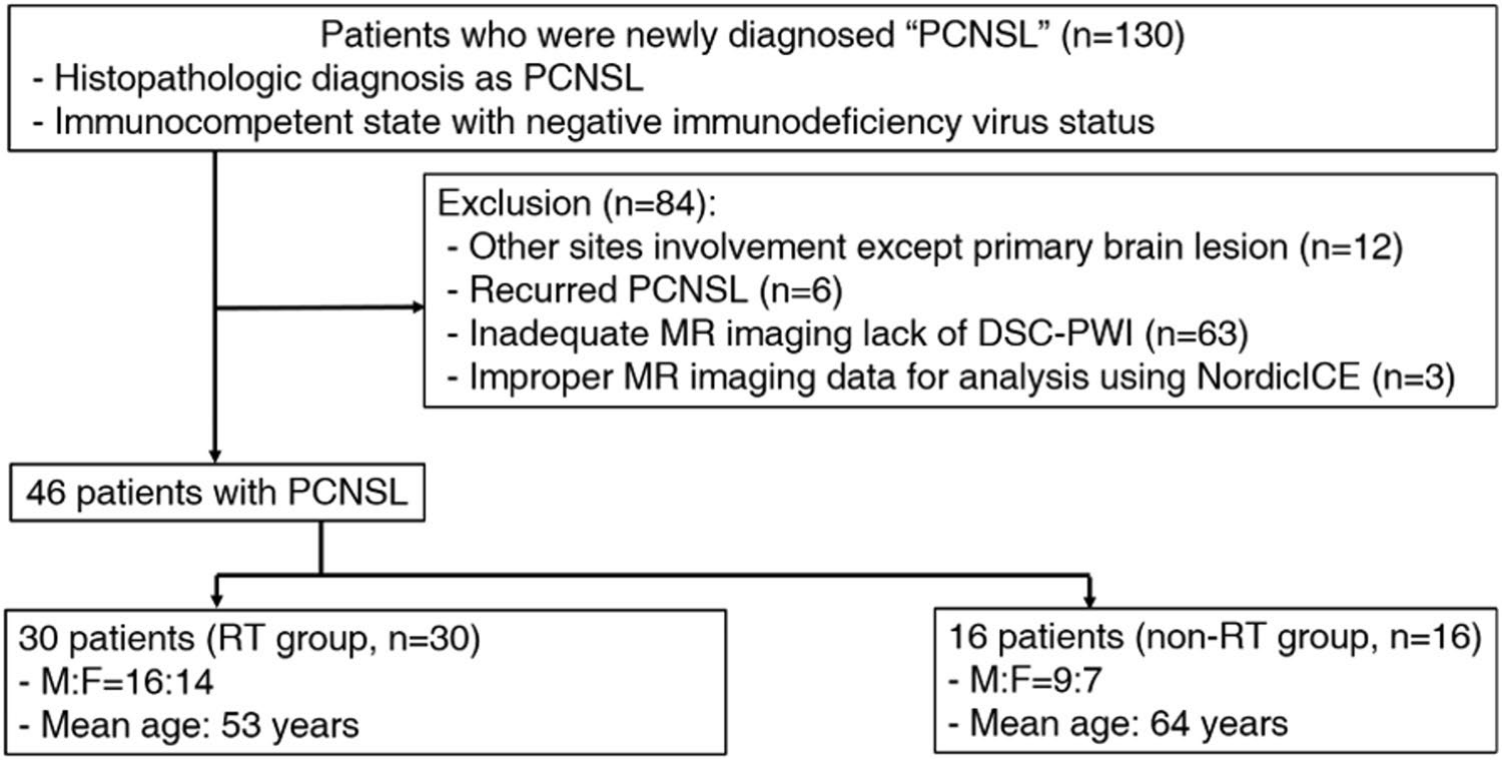
Flowchart for selecting the study population.

Finally, 46 patients treated with chemotherapy were included for this study population (25 men, 21 women; mean age, 56 years; age range, 37–84 years) and were divided into two groups with RT stratification, with combined RT treatment (RT group, n = 30) and without combined-RT treatment (non-RT group, n = 16) (Fig. 5). The RT treatment was decided by neuro-oncology team, which was described in electronic medical record system.

Initial chemotherapy included various regimens as follows: combination of rituximab, methotrexate, procarbazine, and vincristine (n = 33); combination of methotrexate, procarbazine, and vincristine (n = 9); and methotrexate alone (n = 4). All 46 patients received 4–6 cycles of induction chemotherapy with or without RT. The response to therapy was assessed on follow-up MR imaging. A complete response was defined as complete resolution of contrast-enhancing lesions and partial response as an interval decrease in the contrast-enhancing lesions^1^. Whole brain RT (n = 28) and involved filed RT (n = 2) were used as the RT modality for PCNSL patients (18 to 27 Gy to whole brain). All of the 7 patients of the long PFS subgroup and 21 patients of the low PFS subgroup underwent whole brain RT. Other 2 patients of the low PFS subgroup underwent involved field RT only.

### Follow-up and progression free survival with performance status Assessment

The clinical end point measured as the PFS in months was defined as the time from initiation of therapy to the first recurrence. The PFS of each patient was obtained from the medical records of our institution. The ECOG score was assessed after the initial treatment. The average performance score after the initial treatment was 1.41 with a range of from 0 to 5.

### Image acquisition

For each patient, the pretreated MR imaging was performed using 1.5 T (Signa HDxt; GE Medical Systems, Milwaukee, WI) and 3.0 T (Verio; Siemens Medical Solutions, Erlangen, Germany or Biography; Siemens Medical Solutions, Erlangen, Germany or Discovery; GE Medical Systems, Milwaukee, WI or Signa Excite; GE Medical Systems, Milwaukee, WI) scanners which were randomly distributed. The analyzed brain imaging sequences on various MR scanners are presented in Supplementary Table 4. Those sequences included FLAIR, DSC PWI with gadobutrol (Gadovist, Bayer Healthcare, Berlin, Germany), and subsequent contrast-enhanced spin-echo T1 weighted image. For DSC PWI, a single-shot gradient-echo EPI sequences was used during intravenous injection of the contrast agent. For each section, 60 images were obtained at intervals equal to the repetition time. After four to five time points, a bolus of gadobutrol at a dose of 0.1 mmoL/kg of body weight and a rate of 4 ml/sec was injected with an MR compatible power injector (Spectris; Medrad, Pittsburgh, PA, USA). The bolus of the contrast material was followed by a 30 mL bolus of saline, which was administered at the same injection rate.

### Post-processing and histogram analysis

**Figure 6:**
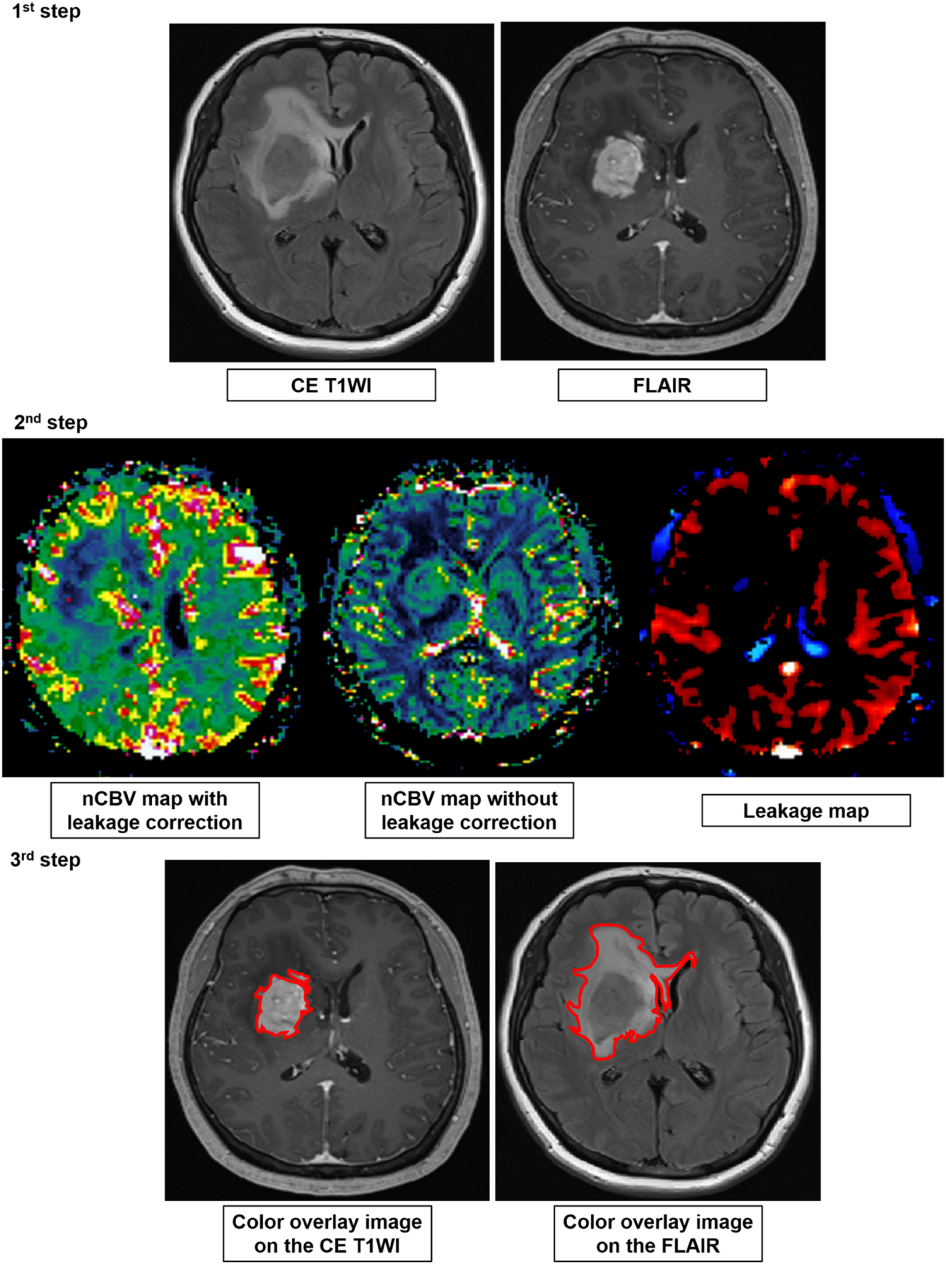
Flow diagram of the histogram analysis. The total VOI was determined with the semiautomatic segmentation method. Based on geometric information stored in the respective data sets of a dedicated imaging processing software, structural MR images such as CE T1WI (1^st^ step, left) and FLAIR (1^st^ step, right) were co-registered with the DSC PWI driven maps, such as the nCBV map with leakage correction (2^nd^ step, left), nCBV map without leakage correction (2^nd^ step, middle), and leakage map (2^nd^ step, right). After acquiring co-registered images on a voxel-by-voxel basis, the total VOIs were drawn (3^rd^ step) on enhancing lesions of CE T1WI and hyperintense lesions of FLAIR. Finally, the overall values for each tumor were obtained by the summation of the histogram parameter values from every plane.

The MR data from the DSC PWI were processed with a dedicated software package (nordicICE; Nordic Imaging Lab, Bergen, Norway). nCBV maps were obtained and applied an established tracer kinetic model for the first-pass data^17,35^. First, realignment was performed to minimized patient motion during dynamic scanning. The gamma-variate function, which is an approximation of the first-pass response at it would appear in the absence of recirculation, was fitted to the 1/T2* curves to reduce the effects of recirculation. Dynamic curves were mathematically corrected to reduce contrast-agent leakage effects^36,37^. After the elimination of recirculation and leakage of the contrast agent, CBV was computed with numeric integration of the curve. To minimize variances in the CBV in an individual patient, the pixel-based CBV maps were normalized by dividing every CBV value in a specific section by the CBV value in the unaffected white matter^38^. Then, maps of nCBVL, nCBVnL, and leakage value (LEAK) were obtained. Six co-registration images in each patient were acquired by co-registration between structural MR imaging, such as CE T1WI and FLAIR and the aforementioned perfusion maps of nCBVL, nCBVnL, and LEAK based on geometric information stored in the respective data sets with the use of the aforementioned image processing software^39^. The differences in the slice thickness between images were automatically corrected by re-slicing and co-registration based on the underlay and structural images. The nCBVL, nCBVnL, and LEAK were displayed as color overlays on the CE T1WI and FLAIR. The total volume of interest (VOI) for the measurable enhancing lesion in each section of the CE T1WI and hyperintensity lesion in each section of the FLAIR were determined by the semiautomatic segmentation method using dedicated software; CE T1WI and FLAIR were used for the structural images. The data acquired from each section were summated to derive the voxel-by-voxel nCBVs for the entire tumor extent of the image using the software (Fig. 6).

Image analysis was performed by one radiologist (Y.S.K. with 1 year of brain MRI experience) blinded to clinical characteristics and outcome, who was supervised an expert neuro-radiologist (S.H.C. with 16 years of experience in neuroradiology) as described below. Histograms of nCBVL, nCBVnL, and LEAK were plotted with each corresponding value on the x-axis, with a bin size of 0.5, while the y-axis was expressed as a total number of pixels. For further quantitative analysis, cumulative histograms were obtained from the previous histograms in which the cumulative number of observations in all bins up to the specified bin was mapped on the y-axis as percentages. The following parameters were derived from the nCBVL, nCBVnL, and LEAK histograms: the mean and 75^th^, 90^th^, and 95^th^ percentile points (the X^th^ percentile point is the point at which X% of the voxel values that form the histogram are found to the left of the histogram)^40^. The abbreviations of imaging parameters used for analysis are shown in Supplementary Table 5.

In addition, the value of total VOIs were obtained from the color overlay image on the CE T1WI and FLAIR, respectively.

### Statistical analysis

All statistical analyses were performed with MedCalc software (v 15.8.0; MedCalc Software, Mariakerke, Belgium). The results with *P* values less than 0.05 were considered significant. The data for each parameter were assessed for normality with the Kolmogorov-Smirnov test. In all tests, *P* values less than 0.05 were considered statistically significant. The clinical characteristics were compared between the RT and non-RT groups using either the Fisher’s exact test or unpaired Student t test.

For the comparison of total VOIs, the mean and 75^th^, 90^th^, and 95^th^ percentiles of the nCBVL, nCBVnL, and LEAK values from histogram analysis of the total VOI, unpaired Student’s t-test or Mann-Whitney test were used. Then, the criteria were determined by the significant difference (*P* < 0.05) for dichotomization of short and long PFS subgroups in both RT and non-RT groups. The ROC curve analysis and multivariate stepwise logistic regression analysis were used to identify independent predictors of the PFS among the aforementioned imaging parameters and its cutoff value. The LOOCV test was performed to evaluate the accuracy of the best predictors.

Kaplan-Meier survival analysis and the log-rank test for the PFS comparison were also performed regarding histogram parameters that had a significant difference between the short PFS and long PFS subgroups, which were dichotomized into two subgroups in both the RT and non-RT groups. In this analysis, patients were defined as having an event if they had been diagnosed with PCNSL progression. Multivariate Cox regression analysis was performed to examine the prognostic significance of the independent predictors.

### Data Availability

All data generated or analyzed during this study are included in this published article and its Supplementary Information files.

### Ethical Approval and Informed Consent

Institutional Review Board approval was obtained and informed consent was waived.

## Electronic supplementary material

Supplementary Tables
