# Supplementary material for: Leakage correction improves prognosis prediction of dynamic susceptibility contrast perfusion MRI in primary central nervous system lymphoma
Source: Sci Rep. 2018 Jan 11;8:456. doi: 10.1038/s41598-017-18901-x (PMC5765049; doi:10.1038/s41598-017-18901-x)
Supplement: Supplementary file 1 — Supplementary Tables [file 41598_2017_18901_MOESM1_ESM.doc]

**Leakage correction improves prognosis prediction of dynamic susceptibility contrast perfusion MRI in primary central nervous system lymphoma**

Original article

Yeon Soo Kim, MD^1^, Seung Hong Choi, MD, PhD (*)^1,2^ , Roh-Eul Yoo, MD^1^, Koung Mi Kang, MD^1^ , Tae Jin Yun, MD, PhD^1^ , Ji-hoon Kim, MD, PhD^1^, Chul-Ho Sohn, MD, PhD^1^ , Sung-Hye Park, MD, PhD^3^ , Jae-Kyung Won, MD, PhD ^3^ , Tae Min Kim, MD, PhD^4^, Chul-Kee Park, MD, PhD^5^, Il Han Kim, MD, PhD^6^

^1^Department of Radiology, Seoul National University Hospital, 101 Daehak-ro, Jongno-gu, Seoul, 03080, Korea

^2^ Department of Radiology, Seoul National University College of Medicine, 103 Daehak-ro, Jongno-gu, Seoul, 110-799, Republic of Korea

^3^ Department of Pathology, Seoul National University College of Medicine, Seoul, Korea

^4^Department of Internal Medicine, Cancer Research Institute, Seoul National University College of Medicine, Seoul, Korea

^5^Department of Neurosurgery, Biomedical Research Institute, Seoul National University College of Medicine, Seoul, Korea

^6^Department of Radiation Oncology, Cancer Research Institute, Seoul National University College of Medicine, Seoul, Korea

Funding: This study was supported by a grant from the Korea Healthcare technology R&D Projects, Ministry for Health, Welfare & Family Affairs (HI16C1111), by the Bio & Medical Technology Development Program of the NRF funded by the Korean government, MSIP (NRF-2015M3A9A7029740), by the Brain Research Program through the National Research Foundation of Korea (NRF) funded by the Ministry of Science, ICT & Future Planning (2016M3C7A1914002), by Creative-Pioneering Researchers Program through Seoul National University (SNU), and by Project Code (IBS-R006-D1).

(*) Address correspondences to Seung Hong Choi, MD, PhD

Department of Radiology, Seoul National University Hospital, 101 Daehak-ro, Jongno-gu, Seoul, 110-744, Republic of Korea

Department of Radiology, Seoul National University College of Medicine, 103 Daehak-ro, Jongno-gu, Seoul, 110-799, Republic of Korea

Center for Nanoparticle Research, Institute for Basic Science (IBS), Seoul 151-742, Republic of Korea

Tel: 82-2-3668-7832; Fax: 82-2-747-7418

E-mail: verocay@snuh.org

**Supplementary table 1.** Comparison analysis for the nCBV value with or without leakage correction co-registered with CE T1WI or FLAIR between the PFS subgroups in the RT group

|  | Short PFS (< 3 years) | Long PFS (≥ 3 years) | *p*-value |
| --- | --- | --- | --- |
| CE T1WI |  |  |  |
| Total VOI mean (95% CI) | 24.6725 (8.5422 – 83.3621) | 18.0478 (15.0151 – 21.8149) | 0.1938 |
| T1 nCBVL mean | 4.1908 | 2.8951 | 0.0466 |
| T1 nCBVL 75% | 5.4409 | 3.8600 | 0.0427 |
| T1 nCBVL 90% | 7.3173 | 5.1105 | 0.0380 |
| T1 nCBVL 95% | 8.9017 | 6.6982 | 0.0640 |
| T1 nCBVnL mean | 3.6450 | 2.3876 | 0.0505 |
| T1 nCBVnL 75% | 4.5533 | 3.0791 | 0.0467 |
| T1 nCBVnL 90% | 7.4712 | 4.8993 | 0.0868 |
| T1 nCBVnL 95% | 6.0285 | 8.9166 | 0.1480 |
| T1 LEAK mean | -0.0293 | -0.3240 | 0.1348 |
| T1 LEAK 75% | 0.2087 | -0.0154 | 0.2742 |
| T1 LEAK 90% | 1.0293 | 0.5660 | 0.1480 |
| T1 LEAK 95% | 1.8674 | 1.1873 | 0.4771 |
| FLAIR |  |  |  |
| Total VOI mean (95% CI) | 112.8772 (33.5856 – 192.1688) | 117.8079 (93.6180 – 141.9978) | 0.8586 |
| FLAIR nCBVL mean | 1.9417 | 1.9989 | 0.7360 |
| FLAIR nCBVL 75% | 3.2854 | 2.6660 | 0.3643 |
| FLAIR nCBVL 90% | 4.8388 | 4.1958 | 0.2112 |
| FLAIR nCBVL 95% | 6.2813 | 5.1593 | 0.2296 |
| FLAIR nCBVnL mean | 1.5277 | 1.4677 | 0.5399 |
| FLAIR nCBVnL 75% | 2.6360 | 2.5150 | 0.7252 |
| FLAIR nCBVnL 90% | 4.5991 | 3.9961 | 0.3390 |
| FLAIR nCBVnL 95% | 5.8122 | 4.8738 | 0.3390 |
| FLAIR LEAK mean | -0.1681 | -0.1142 | 0.8254 |
| FLAIR LEAK 75% | -0.0104 | 0.1608 | 0.3908 |
| FLAIR LEAK 90% | 0.3177 | 0.3641 | 0.7499 |
| FLAIR LEAK 95% | 0.7260 | 0.6650 | 0.9024 |

**Supplementary table 2. Comparison analysis for the nCBV value with or without leakage correction co-registered with CE T1WI or FLAIR between the PFS subgroups in the** non-RT group

|  | Short PFS (< 1 years) | Long PFS (≥ 1 years) | *p*-value |
| --- | --- | --- | --- |
| CE T1WI |  |  |  |
| Total VOI mean (95% CI) | 22.8838 (6.0991 – 44.4593) | 14.7023 (5.4685 – 42.4407) | 1.0000 |
| T1 nCBVL mean | 2.5756 | 3.9443 | 0.0404 |
| T1 nCBVL 75% | 2.9647 | 5.7287 | 0.0115 |
| T1 nCBVL 90% | 3.9888 | 7.9965 | 0.0228 |
| T1 nCBVL 95% | 5.0691 | 8.8436 | 0.0695 |
| T1 nCBVnL mean | 1.5676 | 3.1917 | 0.0857 |
| T1 nCBVnL 75% | 2.7339 | 5.1890 | 0.0614 |
| T1 nCBVnL 90% | 4.4168 | 6.5397 | 0.0877 |
| T1 nCBVnL 95% | 5.9473 | 7.7571 | 0.1588 |
| T1 LEAK mean | 0.0092 | -0.1036 | 0.9374 |
| T1 LEAK 75% | 0.3601 | 0.1384 | 0.7913 |
| T1 LEAK 90% | 0.6590 | 0.8210 | 0.7913 |
| T1 LEAK 95% | 1.2442 | 1.1889 | 0.4273 |
| FLAIR |  |  |  |
| Total VOI mean (95% CI) | 133.7844 (86.3136 – 181.2552) | 153.8893 (76.8951 – 230.8834) | 0.5870 |
| FLAIR nCBVL mean | 1.7594 | 2.0857 | 0.7757 |
| FLAIR nCBVL 75% | 2.3497 | 3.4493 | 0.3154 |
| FLAIR nCBVL 90% | 3.5335 | 5.3739 | 0.1683 |
| FLAIR nCBVL 95% | 4.6763 | 6.6086 | 0.1703 |
| FLAIR nCBVnL mean | 1.7711 | 1.6436 | 0.7260 |
| FLAIR nCBVnL 75% | 2.2538 | 2.9722 | 0.5697 |
| FLAIR nCBVnL 90% | 3.6908 | 4.7882 | 0.5450 |
| FLAIR nCBVnL 95% | 5.0246 | 5.8003 | 0.2817 |
| FLAIR LEAK mean | -0.0749 | -0.0663 | 0.9578 |
| FLAIR LEAK 75% | 0.1498 | 0.1221 | 0.5604 |
| FLAIR LEAK 90% | 0.5498 | 0.3171 | 0.1009 |
| FLAIR LEAK 95% | 0.9435 | 0.5655 | 0.0807 |

**Supplementary table 3. Leave-one-out cross-validation study**

| Group | Model  number | AUC | *p*-value | T1 nCBVL_75%_  cut-off value | Accuracy (%) | Expected  PFS subgroup | Actual  PFS subgroup |
| --- | --- | --- | --- | --- | --- | --- | --- |
| RT | 1 | 0.831 (0.646 – 0.944) | 0.0001 | 5.3250 | 84.09 | long | short ^a^ |
|  | 2 | 0.768 (0.575 – 0.904) | 0.0039 | 5.3250 | 82.61 | long | long |
|  | 3 | 0.769 (0.575 – 0.904) | 0.0039 | 5.3250 | 82.61 | long | long |
|  | 4 | 0.768 (0.575 – 0.904) | 0.0039 | 5.3250 | 82.61 | long | long |
|  | 5 | 0.812 (0.624 – 0.932) | 0.0002 | 5.3250 | 84.09 | long | short ^a^ |
|  | 6 | 0.812 (0.624 – 0.932) | 0.0002 | 5.3250 | 84.09 | long | short ^a^ |
|  | 7 | 0.812 (0.624 – 0.932) | 0.0002 | 5.3250 | 84.09 | long | short ^a^ |
|  | 8 | 0.812 (0.624 – 0.932) | 0.0002 | 5.3250 | 84.09 | long | short ^a^ |
|  | 9 | 0.812 (0.624 – 0.932) | 0.0002 | 5.3250 | 84.09 | long | short ^a^ |
|  | 10 | 0.804 (0.615 – 0.927) | 0.0009 | 5.3250 | 82.61 | long | long |
|  | 11 | 0.805 (0.616 – 0.928) | 0.0003 | 5.3250 | 84.09 | long | short ^a^ |
|  | 12 | 0.805 (0.616 – 0.928) | 0.0003 | 5.3250 | 84.09 | long | short ^a^ |
|  | 13 | 0.819 (0.632 – 0.936) | 0.0002 | 5.3250 | 82.61 | long | long |
|  | 14 | 0.819 (0.632 – 0.936) | 0.0002 | 5.3250 | 82.61 | long | long |
|  | 15 | 0.819 (0.632 – 0.936) | 0.0002 | 4.9128 | 82.61 | short | long ^a^ |
|  | 16 | 0.786 (0.594 – 0.915) | 0.0014 | 5.3250 | 81.82 | short | short |
|  | 17 | 0.786 (0.594 – 0.915) | 0.0014 | 5.3250 | 81.82 | short | short |
|  | 18 | 0.786 (0.594 – 0.915) | 0.0014 | 5.3250 | 81.82 | short | short |
|  | 19 | 0.786 (0.594 – 0.915) | 0.0014 | 5.3250 | 81.82 | short | short |
|  | 20 | 0.786 (0.594 – 0.915) | 0.0014 | 5.3250 | 81.82 | short | short |
|  | 21 | 0.786 (0.594 – 0.915) | 0.0014 | 5.3250 | 81.82 | short | short |
|  | 22 | 0.786 (0.594 – 0.915) | 0.0014 | 5.3250 | 81.82 | short | short |
|  | 23 | 0.786 (0.594 – 0.915) | 0.0014 | 5.3250 | 81.82 | short | short |
|  | 24 | 0.786 (0.594 – 0.915) | 0.0014 | 5.3250 | 81.82 | short | short |
|  | 25 | 0.786 (0.594 – 0.915) | 0.0014 | 5.3250 | 81.82 | short | short |
|  | 26 | 0.786 (0.594 – 0.915) | 0.0014 | 5.3250 | 81.82 | short | short |
|  | 27 | 0.786 (0.594 – 0.915) | 0.0014 | 5.3250 | 81.82 | short | short |
|  | 28 | 0.786 (0.594 – 0.915) | 0.0014 | 5.3250 | 81.82 | short | short |
|  | 29 | 0.786 (0.594 – 0.915) | 0.0014 | 5.3250 | 81.82 | short | short |
|  | 30 | 0.786 (0.594 – 0.915) | 0.0014 | 5.3250 | 81.82 | short | short |
| non-RT | 1 | 0.852 (0.578 – 0.978) | 0.0006 | 4.2243 | 83.34 | short | short |
|  | 2 | 0.893 (0.628 – 0.991) | 0.0001 | 4.2243 | 87.50 | short | long ^a^ |
|  | 3 | 0.821 (0.543 – 0.966) | 0.0047 | 4.2243 | 81.25 | long | long |
|  | 4 | 0.857 (0.584 – 0.980) | 0.0008 | 4.2243 | 87.50 | short | long ^a^ |
|  | 5 | 0.821 (0.543 – 0.966) | 0.0047 | 4.2243 | 81.25 | long | long |
|  | 6 | 0.870 (0.600 – 0.985) | 0.0001 | 3.7458 | 83.34 | long | short ^a^ |
|  | 7 | 0.870 (0.600 – 0.985) | 0.0001 | 4.2243 | 83.34 | short | short |
|  | 8 | 0.821 (0.543 – 0.966) | 0.0047 | 4.2243 | 81.25 | long | long |
|  | 9 | 0.852 (0.578 – 0.978) | 0.0006 | 4.2243 | 83.34 | short | short |
|  | 10 | 0.815 (0.535 – 0.963) | 0.0066 | 4.2243 | 83.34 | short | short |
|  | 11 | 0.815 (0.535 – 0..963) | 0.0066 | 4.2243 | 83.34 | short | short |
|  | 12 | 0.821 (0.543 – 0.966) | 0.0047 | 4.2243 | 81.25 | long | long |
|  | 13 | 0.821 (0.543 – 0.966) | 0.0047 | 4.2243 | 81.25 | long | long |
|  | 14 | 0.821 (0.543 – 0.966) | 0.0047 | 4.2243 | 81.25 | long | long |
|  | 15 | 0.893 (0.628 – 0.991) | 0.0001 | 4.2243 | 07.50 | short | long ^a^ |
|  | 16 | 0.815 (0.535 – 0.963) | 0.0066 | 4.2243 | 83.34 | short | short |

Note. ^a^ The actual PFS subgroup was different from the expected PFS subgroup based on cut-off value calculated using leave-one-out cross-validation study.

**Supplementary table 4. Magnetic resonance scan parameters**

|  | Verio (Siemens) | Discovery MR750W (GE) | DiscoveryBiograph mMR (GE) | Signa Excite (GE) | Signa HDxt (GE) |
| --- | --- | --- | --- | --- | --- |
| Patient number | 23 | 2 | 3 | 1 | 17 |
| Field strength (T) | 3.0 | 3.0 | 3.0 | 3.0 | 1.5 |
| Head coil channel | 32 | 32 | 16 | 8 | 8 |
| DSC-PWI |  |  |  |  |  |
| Repetition time (ms) | 1600 | 1500 | 1500 | 1500 | 1500 |
| Echo time (ms) | 30 | 29.3 | 30 | 40 | 40 |
| Flip angle (°) | 90 | 60 | 90 | 35 | 35 |
| Number of excitation | 1 | 1 | 1 | 1 | 1 |
| Matrix | 128x128 | 100x100 | 128x128 | 128x128 | 128x128 |
| Section thickness (mm) | 6 | 5 | 6 | 5 | 5 |
| Intersection gap (mm) | 0.9 | 1.5 | 1.5 | 1 | 1 |
| Field of view (mm) | 240x240 | 240x240 | 240x240 | 220x220 | 240x240 |
| Scan time | 1 min 44 sec | 1 min 30 sec | 1 min 30sec | 1 min 30 sec | 1 min 30 sec |
| T1 |  |  |  |  |  |
| Repetition time (ms) | 1420 | 8.4 | 1800 | 466.7 | 10.4 |
| Echo time (ms) | 1.9 | 3.2 | 2.49 | 11.0 | 4.7 |
| Flip angle (°) | 9 | 12 | 9 | 69 | 20 |
| Number of excitation | 1 | 1 | 1 | 1 | 1 |
| Matrix | 256x232 | 256x230 | 256x256 | 320x192 | 240x240 |
| Section thickness (mm) | 1 | 1 | 1 | 5 | 1 |
| Intersection gap (mm) | 0 | 0 | 0 | 1 | 0 |
| Field of view (mm) | 249x249 | 256x256 | 240x240 | 220x220 | 240x240 |
| FLAIR |  |  |  |  |  |
| Repetition time (ms) | 5100 | 5124.5 | 9000 | 5000 | 4850 |
| Echo time (ms) | 89 | 92.6 | 97 | 131.1 | 121.9 |
| Flip angle (°) | 16 | 142 | 140 | 90.0 | 90 |
| Number of excitation | 3 | 2 | 1 | 2 | 2 |
| Matrix | 640x384 | 448x448 | 384x209 | 448x256 | 448x256 |
| Section thickness (mm) | 5 | 5 | 5 | 5 | 5 |
| Intersection gap (mm) | 1 | 1 | 1 | 1 | 1 |
| Field of view (mm) | 199x220 | 220x220 | 199x220 | 220x220 | 220x220 |

**Supplementary table 5. Imaging parameter descriptions**

| Imaging Parameter | | Description |
| --- | --- | --- |
| nCBV with leakage correction | | |
| T1 nCBVL mean or X% | Mean or X^th^ percentile of the nCBV with leakage correction of the enhancing portion on the CE T1WI | |
| FLAIR nCBVL mean or X% | Mean or X^th^ percentile of the nCBV with leakage correction of the hyperintensity on the FLAIR | |
| nCBV without leakage correction | | |
| T1 nCBVnL mean or X% | Mean or X^th^ percentile of the nCBV without leakage correction of the enhancing portion on the CE T1WI | |
| FLAIR nCBVnL mean or X% | Mean or X^th^ percentile of the nCBV without leakage correction of the hyperintensity on the FLAIR | |
| Leakage value | | |
| T1 LEAK mean or X% | Mean or X^th^ percentile of the leakage value of the enhancing portion on the CE T1WI | |
| FLAIR LEAK mean or X% | Mean or X^th^ percentile of the leakage value of the hyperintensity on the FLAIR | |
